# Assessment of the State of the Natural Antioxidant Barrier of a Body in Patients Complaining about the Presence of Tinnitus

**DOI:** 10.1155/2018/1439575

**Published:** 2018-10-28

**Authors:** Katarzyna Pawlak-Osińska, Henryk Kaźmierczak, Maria Marzec, Daria Kupczyk, Rafał Bilski, Emilia Mikołajewska, Dariusz Mikołajewski, Beata Augustyńska

**Affiliations:** ^1^Department of Pathophysiology of Hearing and Balance, Ludwik Rydygier Collegium Medicum in Bydgoszcz, Nicolaus Copernicus University, Toruń, Poland; ^2^Department of Otolaryngology and Otolaryngological Oncology Unit with Subunit of Audiology and Phoniatrics, Ludwik Rydygier Collegium Medicum in Bydgoszcz, Nicolaus Copernicus University, Toruń, Poland; ^3^Department of Medical Biology and Biochemistry, Ludwik Rydygier Collegium Medicum in Bydgoszcz, Nicolaus Copernicus University, Toruń, Poland; ^4^Department of Physiotherapy, Ludwik Rydygier Collegium Medicum in Bydgoszcz, Nicolaus Copernicus University, Toruń, Poland; ^5^Neurocognitive Laboratory, Centre for Modern Interdisciplinary Technologies, Nicolaus Copernicus University, Toruń, Poland; ^6^Institute of Mechanics and Applied Computer Science, Kazimierz Wielki University, Bydgoszcz, Poland; ^7^Institute of Physical Education, Kazimierz Wielki University in Bydgoszcz, Poland

## Abstract

**Background:**

Tinnitus is defined as a phantom auditory perception, i.e., sound experience despite the lack of acoustic stimuli in the environment. The aim of this study was to assess the state of the natural antioxidant barrier of a body in patients complaining about the presence of tinnitus.

**Material and Methods:**

The study included a total of 51 patients aged from 20 to 62 years with diagnosed idiopathic tinnitus and 19 healthy subjects as a control group. All patients underwent the audiometric tone test, speech audiometry, distortion otoacoustic emission product testing, study of evoked auditory potentials of short latency, and biochemical analysis of venous blood concerning values of activity or concentration of glutathione, glutathione peroxidase, S-transferase, glutathione reductase superoxide dismutase, malondialdehyde, and ceruloplasmin as the selected parameters of oxidative stress.

**Results:**

Disorders of the auditory pathway were not only limited to the cochlea but also covered its further episodes. Mean values of activity or concentration of the selected parameters of oxidative stress in the study and control groups showed reduced effectiveness of the body's natural antioxidant barrier.

**Discussion:**

Patients complaining about the presence of tinnitus showed reduced effectiveness of the body's natural antioxidant barrier compared to the control group.

**Conclusions:**

The main indication to undertake further research on the functioning of the antioxidant barrier in people suffering from ailments in the form of tinnitus is to determine a suitable therapy aimed at improving the quality of life of these patients, which might be the administration of antioxidant medications.

## 1. Introduction

Oxygen, also called the element of life, is involved in many processes taking place in aerobic organisms. At the same time, it undergoes changes, which result in the production of particles harmful to the body [1].

Oxygen acts as an oxidant. During the reaction with organic compounds, it collects electrons and undergoes a process of reduction. The oxygen molecule undergoes an ultimate four-electron reduction, whereby two molecules of water are created. When the reduction of oxygen molecules is incomplete, reactive oxygen species (ROS) may be formed, which react with cellular components, causing changes in their composition and damaging them [2].

ROS include neutral molecules or ions and oxygen free radicals, i.e., atoms or molecules that have at least one or more unpaired electrons [3, 4]. The main source of ROS in organisms is the mitochondrial respiratory chain and electron transport associated with it [5, 6]. Under homeostasis, ROS play an important role in the metabolism of cells; among others, they are involved in the transmission of signals inside the cell.

In the organism, oxidation reactions overwhelm, and the process is called oxidative stress [7]. The result of its action is damaging DNA as well as changing the structure and function of proteins. [8]. As a result of oxidative stress, lipid peroxidation occurs [9]. This is a process of oxidation of unsaturated fatty acids. The main product of lipid peroxidation is malondialdehyde (MDA) [10].

ROS activity in the body is balanced by antioxidants, substances which inhibit the oxidation degree of the particles and cause their transformation into inactive derivatives. The antioxidants in the human organism belong to the two main groups: endogenic and exogenous. The endogenous antioxidants are enzymes such as superoxide dismutase, catalase, glutathione peroxidase, glutathione S-transferase, and glutathione reductase. The main role in enzymatic antioxidant reactions belongs to the glutathione, which is used as cosubstrate in oxidation and reduction processes. The antioxidant properties of the human organism depend also on the activity of endogenous proteins such as ceruloplasmin. Determination of these parameters is used to evaluate the efficiency of the body in scavenging free radicals. It is also a measure of balance and an indicator of depletion of antioxidants in the body.

The imbalance between ROS and antioxidants leads to many pathological disorders in the human body. Due to the development of oxidative stress, the damage of the inner ear occurs. It leads to the hearing loss and the presence of the tinnitus phenomenon.

Tinnitus is defined by Jastreboff as a phantom auditory perception, i.e., sound experience despite the lack of acoustic stimuli in the environment [11]. Common and basic division classifies tinnitus as objective and subjective [12]. Objective tinnitus, defined as alleged or objective, occurs less often and is associated with the generation of sound by the internal biological origins reaching the ears as a result of conduction tissue. The other type is subjective tinnitus, known as real or subjective, resulting from experiencing phantom sounds, without any known source—heard only by the patient [13, 14]. This phenomenon negatively affects the quality of life of patients, in some cases, to the extent that leads to attempted suicide [15]. Tinnitus is often associated with affective disorders, an example of which may be depression. Studies show that there is a correlation between the severity of symptoms and the patients falling into depression. [16] It is difficult to determine the severity of the disease clearly, because so far, medicine offers no effective diagnostic methods allowing quantitative analysis of the symptom, which is the noise (tinnitus). Measuring tinnitus in practice is based on a subjective assessment of their intensity, frequency, nuisance, location, and masking [17].

Although it is difficult to clearly and selectively establish the cause of the noise, some authors trace the significance of reactive oxygen species in the course of the problem [18, 19]. ROS are formed in the cochlear hair cells exposed to a variety of factors such as cisplatin, salicylates, aminoglycosides, and noise [20–22]. Research confirms that among employees who have a risk factor of occupational noise, statistically significant increase in the prevalence of tinnitus has been detected [23]. Reference is made to beneficial effects of administration of preparations of antioxidants to combat the symptoms of ROS in such ailments [24, 25]. Scientific reports show that primary damage to the inner ear is made as a result of oxidative stress, which results in deterioration of hearing, as well as the occurrence of tinnitus [26]. Aminoglycosides can activate nitric oxide synthase in the tissue of the inner ear which leads to an enhanced efficiency of a reaction catalyzed by the enzyme [27, 28]. Consequently, there is a production of intracellular ROS, apoptosis, and activation of G-proteins and GTPases. These changes result in the activation of protein kinase family, such as MAPK and JNK. The increased kinase activity is accompanied by increased levels of intracellular calcium and the release of cytochrome C from the mitochondria. The described process leads to damage of the mitochondrial membrane by generating pores therein through which cytochrome C flows out. The trail of activation of caspases 3 and 8 is activated. Degradation of the cytoskeleton and chromatin follows. Additionally, calpain cascade is activated. The consequence of this process is damage and destruction of hair cells of the inner ear [29, 30]. The theory presented by Elgoyhen et al. indicates the possibility of distributed damage of the neuron network of the central nervous system, responsible for the registration of sound stimuli. However, the question of whether this damage would be a beginning of noise or a secondary consequence of the disintegration of the receptor of the cochlea remains unexplained. [31, 32]. Reports on increased oxidative activity in venous blood of brain circulation compared to peripheral blood in patients with tinnitus may be a confirmation of the fact of multifocal damage of nerve cells of the central nervous system, appearing not only in the inner ear structures [28, 33].

The essence of the research on the phenomenon of oxidative stress in patients with tinnitus is to search for specific, causal treatment of this ailment, which could be the administration of antioxidant medications.

The aim of this study was to assess the state of the natural antioxidant barrier of a body in patients complaining about the presence of tinnitus.

## 2. Material and Methods

The study included a total of 32 patients aged from 20 to 62 years (mean age: 54.2 years) with idiopathic tinnitus, who were diagnosed with tinnitus in the Department of Otolaryngology and Otolaryngological Oncology Unit with Subunit of Audiology and Phoniatrics of Nicolaus Copernicus University Collegium Medicum in Bydgoszcz. Idiopathic tinnitus was characterized by

1. volume from 10 to 110 dB, mean 58.2 dB
2. frequency from 500 to 8000 H, mean 3370 Hz

All patients underwent the tone audiometric test (audiometer, Interacoustics), speech audiometry (audiometer, Interacoustics), distortion otoacoustic emission product testing (camera, Madsen), and the study of evoked auditory potentials of short latency—BERA (camera, Synapsys).

The control group consisted of 19 healthy subjects (recruited among acquaintances) aged 20 to 60 years (mean age: 49.2), who were not complaining of any audiological problems, well communicating by hearing, not receiving any chronic medication, and defining their physical and mental health as well-being according to the definition of good health by WHO.

The material for analysis was venous blood collected in an amount of approx. 8 ml of the antecubital vein into lithium heparin tubes and tubes without an anticoagulant. Blood samples were collected at 8.00. Then, collected material was transported to the Department of Biochemistry of Nicolaus Copernicus University Collegium Medicum in Bydgoszcz. Tests were carried out on the same day, within approx. 1 hour of material collection. On the basis of own studies, no statistically significant differences in hematocrit between the study and the control group were noted. From the blood drawn into tubes without an anticoagulant (approx. 3 ml), serum was obtained by centrifugation of the material over 5 min at 5000 ×g, and then it was transferred to Eppendorf tubes and frozen at −80°C. The prepared serum was stored to determine the activity of the oxidase ceruloplasmin (Cp). Before preparing the hemolysate, 500 *μ*l of blood was collected to determine the levels of glutathione (GSH) in the erythrocytes; the remaining aliquot of blood (approx. 5 ml) was centrifuged to obtain plasma, wherein the concentration of nitrate/nitrite was determined. The remaining cells were used for the preparation of the hemolysate, wherein the malondialdehyde concentration (MDA) and the activity of the enzymes, glutathione peroxidase (cGPx), glutathione S-transferase (GST), and superoxide dismutase (SOD-1), were determined. The concentration of reduced glutathione (GSH) was assayed using the Beutler method [34]. The principle of this method is based on the reaction of reduction of the disulfide compound—dithio-bis-2-nitrobenzoic acid (DTNB)—by compounds containing sulfhydryl groups. In the blood, free sulfhydryl groups unrelated to proteins derived almost only from GSH. The product of the described reaction is a compound of yellow color. Color density was measured at 412 nm. The calculations used the molar absorption coefficient, which, when attached to the mentioned wavelength, is equal to 13.6 mol^−1^ × l × cm^−1^. The results were expressed in mmol/l. The coefficient of variation for this method was 2.4%.

The activity of glutathione peroxidase (GPx) in erythrocytes was assayed by a two-stage Paglia and Valentine method [35]. In the first stage, GPx reacts with tert-butyl peroxide and reduced glutathione (GSH). The product of this reaction is glutathione disulfide (GSSG). The second stage involves the action of glutathione reductase (GR) reducing GSSG to GSH with the participation of NADPH + H^+^ as a regulator. NADPH oxidation results in a reduction in absorbance at a wavelength of 340 nm, which is measured spectrophotometrically. CGPx activity was calculated based on the loss of the reduced form of coenzyme in time (test Wartburg). In the calculations, millimolar absorption coefficient for NADPH at 340 nm, which is equal to 6.22 mmol^−1^ × l × cm^−1^, was used. The results were expressed in U/g Hb, where 1 *μ*mol oxidation of NADPH in one minute at *T* = 25°C was adopted as a unit of enzyme activity. The coefficient of variation for this method was 2.9%.

Determination of glutathione S-transferase (GST) activity in RBCs was performed according to the method of Habig and Jakoby [36]. In this method, there is a decrease in absorbance (which is measured at a wavelength of 340 nm) due to the formation of a conjugate of glutathione (GSH) with 1-chloro-2,4-dinitrobenzene (CDNB). The decrease in absorbance is proportional to the glutathione S-transferase activity. The GST activity assay was carried out in the presence of phosphate buffer and CDNB. The results were expressed in nmol CDNB-GSH/mg Hb/min.

Superoxide dismutase (SOD-1) activity in RBCs was determined using the Misra and Fridovich method, which is based on the inhibition of adrenaline oxidation reaction by superoxide dismutase at pH 10.2. [37]. The increase in absorbance was measured at a wavelength of 480 nm. It is proportional to the increase in the concentration of oxidation products of adrenaline. The activity of SOD-1 was expressed in U/g Hb. The amount of enzyme which inhibits the oxidation of adrenaline 50% was adopted as a U unit. The coefficient of variation for this method is 6.3%.

The concentration of malondialdehyde (MDA) in the erythrocytes was determined by the Placer et al. method, which is based on the reaction of a thiobarbituric acid and certain products of lipid peroxidation, mainly MDA, in an acidic environment and at elevated temperature [38]. This reaction produces a colored product, the color intensity of which was measured at a wavelength of 532 nm. In the calculations, the millimolar absorption coefficient of 156 mmol^−1^ × l × cm^−1^ was used. The result was expressed in mmol/g Hb. The coefficient of variation for this method was 3.5%.

The concentration of nitric oxide was determined using the indirect method according to Marlett, determining the concentration of nitrate/nitrite in plasma. The method is based on the reaction between the nitrate anion and anion from N-(1-naphthyl)ethylenediamine, in the sulfanilic acid environment (Griess reaction) [39]. This reaction produced a colored complex whose absorbance is measured at a wavelength of 545 nm. It is directly proportional to the concentration of nitrates and nitrites in the studied sample. The result was expressed in *μ*mol/l.

Ceruloplasmin oxidase activity was determined using the method of Ravin [40]. The principle of the method is based on oxidation of substrate p-phenylenediamine (PPD) by ceruloplasmin at a final purple-colored product. Absorbance measurement was made at a wavelength of 530 nm. This product is so-called “the principle of Bandrowski” (product formed from three molecules of the substrate). Results were expressed in international units.

All the data in this study were collected and stored using the MS Access 2013 software, where the available mean, median, minimum value (min), the maximum value (max), and standard deviation (SD) were calculated to show the results of this study. The Shapiro-Wilk test was used as a powerful normality *test*. Parametric Student's *t*-test and nonparametric Wilcoxon's test were used to compare scores. Statistical analysis was performed using the IBM SPSS Statistics. The difference was statistically significant at *p* < 0.05.

This study was conducted in accordance with the Declaration of Helsinki and the guidelines for Good Clinical Practice (GCP). Freely given written informed consent was obtained from every patient prior to the study.

## 3. Results

The results of tone audiometry are presented in Table 1. The hearing loss was evident from the frequency of 6000–8000 Hz in the majority of respondents (45 patients); it was larger on the left side but generally slightly deviated from the standard curves in the age groups of the Polish population.

Speech audiometry showed that all patients reached 100% when distinguishing words with the exception of one patient with a distinction loss of 20% in the left ear. The threshold for distinguishing in the whole study group was 20 to 80 dB (mean: 34.8 dB) in the right ear and 20 to 90 dB (mean: 44.3 dB) in the left ear. Acoustic otoemissions objectively assessing the function of the cochlea were affected in all tested ears, with the exception of 4 right and 3 left ears. Most frequently, reduced acoustic energy at frequency of 1000 Hz in the right ear (19 patients) and at frequencies of 1000 Hz (18 people) and 4000 Hz in the left ear (16 patients) was reported. Pathological records of nerve impulse conduction from the cochlea through the VIII nerve to the brainstem and third-order auditory neuron (BERA) were obtained from 14 patients, including both ears in 5 patients and one ear in 9 patients. Disorders applied to the prolonged latency of waves I, III, and V and increased interlatency of waves I–III, III–V, and I–V. Prolonged latencies of wave I were stated in 8 ears, wave III in 7 ears, and wave V in 6 ears. Increased intervals of waves I–III were present in 7 ears, wave III–V in 6 ears, and the waves I–V in one ear. On this basis, it can be concluded that disorders of the auditory pathway are not only limited to the cochlea but also covered its further episodes.

Table 2 shows the mean values of activity or concentration of the selected parameters of oxidative stress in the study and control groups. The average concentration of reduced glutathione in erythrocytes in the study group was higher than in the control group. Statistical significance of differences within the variable is *p* = 0.000. Glutathione peroxidase activity was significantly lower in the study group compared with the control group. The level of statistical significance is *p* = 0.026, which meets the criterion of significance (*p* < 0.05). Also, lower erythrocyte glutathione peroxidase activity in the study group compared with the control group was noted. Statistical significance for the determination of this parameter was *p* = 0.000. A higher activity of glutathione S-transferase in patients with symptoms of tinnitus as compared to healthy subjects (*p* = 0.000) was observed. On the basis of statistical analysis, differences in the activity of superoxide dismutase between the study group and the control group were demonstrated. The activity of this enzyme was lower in the study group than in the control group (*p* = 0.000). Statistically significant differences in the concentrations of malondialdehyde between the groups were demonstrated. The concentration of MDA in the study group was higher than in the control group (*p* = 0.005). Nitric oxide concentration in plasma (measured indirectly based on the concentration of nitrate/nitrite) was significantly higher in the study group compared with the control group (*p* = 0.008). No significant differences in the activity of ceruloplasmin oxidase between the study group and the control group (*p* = 0.188) were noted. There were no statistically significant differences in hematocrit values between patients with ailments in the form of tinnitus compared to the healthy controls (*p* = 0.153).

## 4. Discussion

Reactive oxygen species are molecules which are formed in many biological processes. When they are released in physiological amounts, they act as mediators and regulators. That way, they ensure proper functioning of the cells [41, 42].

Superoxide dismutase (SOD-1) converts oxygen radicals into molecular oxygen and hydrogen peroxide. Its activity is inhibited by H_2_O_2_ produced in this process [43, 44]. In the study by Zhang et al., a decrease in erythrocyte superoxide dismutase activity in patients with deterioration of hearing was observed, and some of them complained of feeling the tinnitus [45]. Also in our study, we observed a statistically significant decrease in the activity of this enzyme in the red blood cells of people complaining of discomfort in the form of tinnitus. This is probably the result of a high content of superoxide anion, which inhibits activity of SOD-1. cGPx catalyzes the reaction of reduction of hydrogen peroxide under the influence of glutathione (GSH). The activity of glutathione peroxidase is inhibited by high levels of superoxide anion, while growth in its activity indicates increased activity of the antioxidant barrier system [46, 47]. In our study, we found a statistically significant decrease in the activity of glutathione peroxidase in erythrocytes in patients suffering from ailments in the form of tinnitus. The activity of superoxide dismutase and glutathione peroxidase in the erythrocytes in the study group decreased, which may indicate an impaired functioning of the antioxidant barrier within the red blood cells. In turn, in their studies, Neri et al. showed, among others, elevated levels of glutathione peroxidase in patients with acute tinnitus. The authors also showed elevated levels of malondialdehyde, 4-hydroxynonenal, and myeloperoxidase and decreased levels of nitric oxide. Based on the results, researchers concluded that this condition may contribute to the overall dysfunction of endothelia, inducing the microvascular changes in the inner ear [33]. The study by van de Heyning et al. confirmed the efficacy of the intratympanic AM-101 (0.27 or 0.81 mg/ml) in the treatment of tinnitus arising from cochlear glutamate excitotoxicity reflected in patient-reported outcomes [48]. Another parameter for assessing disorders of the antioxidant barrier in erythrocytes is to assay the activity of glutathione S-transferase (GST) [49]. In a study of Liu et al., patients with tinnitus who were administered formulation of *Ginkgo biloba* were compared with the patients receiving placebo. In the group of people who had not received the medication, GST values were increased [50]. Our study showed a statistically significant increase in the activity of GST within erythrocytes of people in the study group when compared to the control group. This is probably due to a reduction in the efficiency of glutathione peroxidase, which results in the induction of GST activity. In the plasma of patients in the study group, a statistically significant decrease in activity of cGPx was demonstrated compared with the plasma of the control group. Also, in the study of Wang and Xie, regarding the evaluation of aging of a hearing organ, a reduced level of cGPx in the plasma of patients complaining of hearing problems was observed [51]. This is probably due to the fact that peroxidase plasma overreacted by reducing hydrogen peroxide [52–54]. In our study, statistically significant higher levels of glutathione in the plasma of subjects from the study group compared to the control group were observed. Similar results in patients with tinnitus were obtained by Liu et al. [55]. However, in their research, Gil et al. demonstrated decreased levels of glutathione among the elderly affected by tinnitus [56]. In people with Meniere's disease, in whom one of the main symptoms is tinnitus, often of a permanent nature, Calabrese et al. observed a decreased level of GSH [57]. A ceruloplasmin, which is a plasma protein, plays a special role in the capture of ROS. It has the ability to bind copper ions, thus preventing the formation of hydroxyl radical from hydrogen peroxide [58]. It has been shown that in oxidation processes, which involve ceruloplasmin, there is no production of harmful ROS or hydrogen peroxide [59, 60]. In our study, no statistically significant differences in the activity of oxidase ceruloplasmin between the study and control groups were demonstrated. This may be due to the fact that ceruloplasmin is an acute-phase protein, and thus, changes in this parameter cannot be attributed to the direct evaluation of the body's antioxidant barrier.

Antioxidant mechanisms are divided into enzymatic and nonenzymatic [61]. The nonenzymatic antioxidant factor is nitric oxide, which plays an important role in the regulation of vascular tone, catches ROS, and protects against lipoperoxidation [62]. In our study, we measured the ratio of nitrite to nitrate concentration, which allowed the assessment of the metabolism of nitric oxide. A significant increase in the metabolism of nitric oxide in the blood of the subjects compared to the controls was noted. However, Coomber et al. in their study conducted in guinea pigs did not obtain statistically significant variability of the level of nitric oxide [63]. In patients complaining of occurrence of symptoms in the form of tinnitus, disturbances in lipid balance may occur with a lack of unsaturated fatty acids that bind to reactive free radicals. The result of damage caused by oxidative stress can be measured via measurement of the concentration of thiobarbituric acid derivatives [64]. For this purpose, malondialdehyde (MDA) is most frequently used. A statistically significant increase in the concentration of this compound is indicative of impaired antioxidant barrier function and consequently permanent damage caused by reactive oxygen species. Studies conducted by Savastano et al. demonstrated statistically significant increase in malondialdehyde concentration in the serum of patients suffering from tinnitus [65]. Also in our study, a significantly higher concentration of MDA was demonstrated among the study subjects compared with the control group. This is probably a result of a reduced effectiveness of the body's natural antioxidant barrier. Changes in the metabolism of both nitric oxide and malondialdehyde may indicate the inefficiency of the natural antioxidant barrier in patients suffering from tinnitus.

An interesting aspect of the characteristics of people with tinnitus is typically abnormalities of lipid balance with deficiency of unsaturated fatty acids that bind to reactive free radicals. Lack of internal mechanisms regulating this abnormality leads to the implementation of external control of antioxidant drugs. Such attempts to treat, among others, Meniere's diseases show improvement in the well-being of patients [66]. It is difficult to find objective evidence of the effectiveness of this type of therapy in idiopathic tinnitus. Savastano et al. [65] recorded subjective improvement in VAS with a subjective reduction in the volume of noise with no reflection in the audiogram. Another theory presented by Menéndez et al. describes treatment using pure oxygen in the form of injection or oxygen hyperbarics. In 50 patients who used injections of oxygen in the artery area, a decrease in the severity of vertigo and nystagmus and hearing impairment was observed, while the relatively smallest, although significant, effect was achieved in the case of tinnitus [67]. The study by Fujimura et al. [68] showed the hearing improvement rate was significantly higher in the HBO group than in the steroid group (51.1 ± 7.0% vs. 27.1 ± 7.8%) in patients with initial hearing levels of ≥80 dB. On the other hand, Desloovere noted that oxygen hyperbarics seem to be more effective in acute incidents of noise than in the treatment of chronic noise [69]. It is difficult to assess the effectiveness of this method in comparison with the subjective feeling of the annoyance of tinnitus [70]. A low percentage (approx. 3%) of patients who report a complete disappearance of tinnitus after oxygen therapy seem to indicate that the role of oxidative stress in the etiology of tinnitus is still unclear. It might be conditioned by the lack of standardization of research material and the lack of objective control after the treatment. A similar role is probably played by the lack of standardization of therapy (type and dose of medications), which, in turn, points to a need for work on the experimental damage of the inner ear model by reactive oxygen molecules. Perhaps the reports of the damaged cochlea and stria vascularis should be extended by biochemical observations of further components of the auditory pathway, especially due to the coexistence of electrophysiological abnormalities of both the cochlea and over cochlea location in patients with tinnitus classified as idiopathic, observed in our work. Due to the fact that the oxidative stress is underlying many diseases, research in this area should be continued [71].

## 5. Conclusions

Our results contribute to the general knowledge about patients with tinnitus classified as idiopathic, observed in our work. Patients complaining about the presence of tinnitus showed reduced effectiveness of the body's natural antioxidant barrier compared to the control group. Obtained results suggest expanding the research in the area of the role of oxidative stress in tinnitus. The results of a larger study may be used to determine a suitable therapy aimed at improving the quality of life of these patients, which might be the administration of antioxidant medications.

## Figures and Tables

**Table 1:** Results of tone audiometry in patients with tinnitus.

Hearing loss for particular frequencies	Frequency (Hz)						
	250	750	1000	2000	4000	6000	8000
Mean hearing loss in the right ear (dB)	21.9	22.9	24.4	26.7	35.2	44.0	48.7
Mean hearing loss in the left ear (dB)	29.0	30.8	30.8	38.5	51.9	60.6	59.2

**Table 2:** Mean values of activity or concentration of the selected parameters of oxidative stress in the study and control groups.

Parameter	Study group (*n* = 32)		Control group (*n* = 19)		*p* value
	Mean	Standard deviation	Mean	Standard deviation	
Hematocrit	41.991	3.193	43.342	3.253	0.153
GSH (mmol/l)	2.566	0.278	2.208	0.162	0.000
GPx plasma (U/g Hb)	220.719	30.77	246.047	48.289	0.026
GPx RBC (U/g Hb)	14.761	2.611	18.911	2.257	0.000
GSTRB (nmol CDNB-GSH/mg Hb/min)	3.222	0.55	2.55	0.402	0.000
SODRBC (U/g Hb)	2479.06	236.812	2805.26	184.771	0.000
MDARBC (mmol/g Hb)	0.276	0.022	0.253	0.034	0.005
NO_2_^−^/NO_3_^−^ (*μ*mol/l)	1.466	1.53	0.812	0.635	0.008
CP (IU)	1166.638	384.075	1340.358	542.449	0.188

## Data Availability

The data used to support the findings of this study are included within the article.
